# Indiana State Dental Association—Fifteenth Annual Session

**Published:** 1873-11

**Authors:** B. P. McDonald


### INDIANA STATE DENTAL ASSOCIATION—FIFTEENTH ANNUAL SESSION.

Met at Templars' Hall in the city of New Albany, June 3rd, 1873, at 3 o'clock P. M.

In the absence of President Richardson, Dr. P. G. C. Hunt, of Indianapolis, was chosen President, *pro tem*; Secretary

McDonald being absent, Dr. S. B. Brown, of Fort Wayne, was selected as Secretary, *pro tem.*

On motion, the reading of the minutes of last meeting was dispensed with ; President Hunt appointed the following gentlemen as censors : Drs. Merrit Wells, W. L. Heiskell and W. E. Driscoll.

The report of the Committee on Dental Law was received, and the Committee discharged.

The Board of Censors reported the following gentlemen as suitable candidates for memberhip in the State Association.

Dr. V. D. Jackson, Jeffersonville ;
" A. Lanning, Salem ;
" W. C. Knapp, Fort Wayne ;
" P. T. Green, New Albany ;
" J. C. Henry "

All of whom were elected to membership by separate ballots.

A letter was read by Dr. John F. Johnston, from Prof. Joseph Richardson, expressing his regret in being detained by sickness in his family. The letter was accepted, and the Secretary instructed to forward a letter of sympathy to him.

The Association then proceeded to the election of officers, with the following result:

*President*, Dr. Merrit Wells, of Indianapolis ;
1*st Vice President*, Dr. Seneca B. Brown, Fort Wayne ;
2*nd* " " Dr. W. E. Driscoll, Bedford ;
*Secretary*, B. P. McDonald, Goshen ;
*Treasurer*, John F. Johnston, Indianapolis ;

President Hunt appointed Drs. Johnston and Heiskell to escort the newly elected President to the chair, who made a brief speech in acknowledgement of the honor conferred.

President Wells appointed the following members to act as Executive Committee for the ensuing year :

Dr. P. G. Hunt, of Indianapolis ;
Dr. Joseph Richardson, Terre Haute ;
Dr. I. Knapp, Fort Wayne ;

The following resolution was presented and unanimously adopted :

*Resolved*, That the Indiana State Dental Association tender its grateful acknowledgment to Dr. S. S. White, for his individual effort to have righted what they conceive to be a great wrong, in the matter of the Gardner Appeal Case.

At this juncture a committee from the Kentucky State Dental Association was received, from whom an invitation was accepted to adjourn, to meet with the Kentucky Association, on Wednesday, and continue with it during the session of the Association.

The following members of the profession from Louisville were elected honorary members : Drs. W. G. Rediman, Chas. Dunn, W. M. Rogers, J. B. McCarthy, L. W. Weld and J. W. Grant.

On motion, the Association adjourned to meet in Louisville, on Wednesday morning at nine o'clock.

MORNING SESSION, SECOND DAY.

Pursuant to adjournment the Association met in the Hall of the School Board, in the city of Louisville, at half-past 10 o'clock, A. M.

Whereupon the two Associations went into joint session.

The Indiana State Dental Association receiving an address of welcome from Dr. Goddard, President of the Kentucky State Dental Association, exhibiting a true fraternal spirit, which cannot but advance the best interests of both Associations for all time to come, and with clasped hands across the river, march forward to newer and grander achievements.

In response, Dr. Merrit Wells returned thanks in the name of the members of the Indiana State Dental Association he represented, to the Kentucky Association for the kindness and fraternal feeling which had been shown toward them, which they would not fail to reciprocate when the occasion offered. They had come to seek knowledge and light with their Kentucky brethren; although separated by geographical lines, they were united in bonds of fraternity and affection, and hoped they should ever continue to be so.

As the Secretary of the Kentucky State Dental Association has reported the action of the joint session upon the various subjects discussed, and the same published, I will not occupy your valuable space by repetition.

The Indiana State Dental Association adjourned to meet at Indianapolis on the first Tuesday in June, 1874.

B. P. Mc [illegible]onald, *Secretary*

